# Comparative interactomes of HSF1 in stress and disease reveal a role for CTCF in HSF1-mediated gene regulation

**DOI:** 10.1074/jbc.RA120.015452

**Published:** 2020-11-24

**Authors:** Eileen T. Burchfiel, Anniina Vihervaara, Michael J. Guertin, Rocio Gomez-Pastor, Dennis J. Thiele

**Affiliations:** 1 Department of Biochemistry, Duke University School of Medicine, Durham, North Carolina, USA; 2 Department of Pharmacology and Cancer Biology, Duke University School of Medicine, Durham, North Carolina, USA; 3 Department of Molecular Biology and Genetics, Cornell University, Ithaca, New York, USA; 4 Department of Biochemistry and Molecular Genetics, University of Virginia, Charlottesville, Virginia, USA; 5 Department of Molecular Genetics and Microbiology, Duke University School of Medicine, Durham, North Carolina, USA

**Keywords:** heat shock transcription factor 1 (HSF1) regulation, immunoprecipitation mass spectrometry, protein interaction, gene repression, striatal transcription, acute and chronic stress response, CCCTC-binding factor (CTCF), CBY1, chibby family member 1, CTCF, CCCTC-binding factor, DMEM, Dulbecco's modified Eagle's medium, FBS, fetal bovine serum, HC, high confidence, HEK, human embryonic kidney, HD, Huntington's disease, HSE, heat shock element, HSF1, heat shock transcription factor 1, IP, immunoprecipitation, LC, low confidence, mHtt, mutant Htt, NEMF, nuclear export mediator factor, PCA, principal component analysis, polyQ, polyglutamine, QC, quality control, SMC6, structural maintenance of chromosomes protein 6, SNX9, sorting nexin-9, TC-1, thyroid cancer-1, XRCC5, X-ray repair cross complementing 5

## Abstract

Heat shock transcription factor 1 (HSF1) orchestrates cellular stress protection by activating or repressing gene transcription in response to protein misfolding, oncogenic cell proliferation, and other environmental stresses. HSF1 is tightly regulated via intramolecular repressive interactions, post-translational modifications, and protein-protein interactions. How these HSF1 regulatory protein interactions are altered in response to acute and chronic stress is largely unknown. To elucidate the profile of HSF1 protein interactions under normal growth and chronic and acutely stressful conditions, quantitative proteomics studies identified interacting proteins in the response to heat shock or in the presence of a poly-glutamine aggregation protein cell-based model of Huntington's disease. These studies identified distinct protein interaction partners of HSF1 as well as changes in the magnitude of shared interactions as a function of each stressful condition. Several novel HSF1-interacting proteins were identified that encompass a wide variety of cellular functions, including roles in DNA repair, mRNA processing, and regulation of RNA polymerase II. One HSF1 partner, CTCF, interacted with HSF1 in a stress-inducible manner and functions in repression of specific HSF1 target genes. Understanding how HSF1 regulates gene repression is a crucial question, given the dysregulation of HSF1 target genes in both cancer and neurodegeneration. These studies expand our understanding of HSF1-mediated gene repression and provide key insights into HSF1 regulation via protein-protein interactions.

Organisms are constantly challenged with adapting to stressful conditions such as protein misfolding, inflammation, environmental toxicants, increased temperature, and rapid cell proliferation. A crucial player in the cellular stress response is heat shock transcription factor 1 (HSF1), a regulator of stress-protective gene transcription (1, 2, 3, 4). In normal cells in the absence of acute stress, the majority of HSF1 is maintained in an inactive, monomeric state and resides predominantly in the cytoplasm (5, 6). This basal repression of HSF1 is achieved through protein interactions including a multichaperone complex (7, 8, 9, 10, 11, 12), proteins that post-translationally modify HSF1, and repressive intramolecular interactions between leucine zipper regions (13, 14, 15, 16, 17). HSF1 is activated in response to stressful conditions through oligomerization and is retained in the nucleus where it binds heat shock elements (HSEs) adjacent to target genes or in distal regulatory elements to promote cell survival through several mechanisms (1, 2). Genes activated by HSF1 encode protein chaperones, the ubiquitin proteasome degradation machinery, cell cycle determinants, transcriptional regulators, and many other proteins involved in diverse processes (3). A more recently explored facet of HSF1 biology is its role in repressing gene transcription, including those involved in apoptosis, inflammation, and transcription (18, 19, 20, 21, 22).

Although HSF1 is primarily cytosolic and inactive in the absence of stress, a small fraction of HSF1 remains in the nucleus and binds to target genes in the absence of acute stress (23, 24). The characterization of HSF1 direct target genes in basal conditions and in response to acute stress, chronic protein misfolding diseases, or in cancer cells reveals cell-context-specific sets of HSF1-regulated genes, some of which contribute to pathogenesis (24, 25, 26, 27, 28). How HSF1 protein regulators are altered to influence different disease signatures is not well understood.

Owing to the central role HSF1 plays in coping with stresses that disrupt protein homeostasis, HSF1 is of great interest in neurodegenerative diseases that arise from chronic protein misfolding including Alzheimer's disease, Parkinson's disease, and Huntington's disease (HD) (5, 29, 30, 31, 32, 33). In HD, a CAG triplet nucleotide expansion in exon 1 of the Huntingtin gene results in a pathogenic polyglutamine (polyQ) expansion in the Htt protein (mutant Htt, mHtt) (34). mHtt protein aggregates in complexes with cellular components including cell signaling proteins, transcription factors, and other key regulatory proteins, disrupting cellular function and ultimately increasing the propensity for apoptosis (35, 36). The protein misfolding and cellular dysfunction is further exacerbated by reduced levels of protein quality control (QC) components, including protein chaperones and the protein degradation machinery, which aid in maintaining protein homeostasis. Consequently, activation of cytoprotective protein chaperones such as Hsp70 and Hsp40, the TRiC chaperonin subunits, and the protein degradation machinery alleviates protein misfolding and augments aggregate clearance in HD (37, 38, 39, 40, 41). Furthermore, activation of multiple chaperone systems via HSF1 is more efficacious than a single chaperone (37, 38, 39, 40, 41) as HSF1 simultaneously elevates levels of protein QC components and regulates target genes to promote protein homeostasis and cell survival; thus, HSF1 activation is a promising point of therapeutic intervention (1, 3, 5, 33, 42, 43, 44). However, in response to mHtt, this stress-protective transcription factor is aberrantly degraded, its target gene expression blunted, and its genome-wide binding dramatically altered, raising questions of how HSF1 is dysregulated in HD (28, 29, 31, 33). A more detailed understanding of the regulation of HSF1 in unstressed cells, the dysfunctional regulation of HSF1 in HD, and how this compares with the acute stress of heat shock (HS) may offer new insights into HSF1 regulation and its contribution to disease.

Previous studies conducted in HD cellular and mouse models revealed that the impairment of HSF1 arises, in part, from inappropriate protein interactions that result in HSF1 degradation (33). In HD, elevated expression of protein kinase CK2α' and E3 ligase component FBXW7 promote the phosphorylation-dependent degradation of HSF1 (33). In contrast to the elevated HSF1 degradation in HD, compromised FBXW7 function in cancers impairs HSF1 degradation, giving rise to increased HSF1 protein levels that support malignancy (45, 46, 47, 48).These and other studies demonstrate the importance of protein-protein interactions in modulating HSF1 nuclear retention, DNA binding, activation or repression of target genes, and degradation (8, 10, 22, 49, 50, 51, 52). For instance, mitochondrial single-stranded DNA-binding protein is crucial for the activation of some HSF1 target genes in response to elevated temperatures (51). BCL2-associated athanogene 3 (BAG3) regulates HSF1 nuclear retention during heat stress (49), whereas RPA70 facilitates basal HSF1 binding at the Hsp70 locus by recruiting histone chaperone FACT (50). In addition to illuminating regulatory mechanisms imposed on HSF1 by interacting proteins, the study of other protein interactors has revealed roles for HSF1 in new pathways, including DNA repair (53, 54), metabolism (55), and the mitochondrial unfolded protein response (56). Taken together, these and other studies demonstrate how protein regulators of HSF1 can significantly alter HSF1 activity, function, and degradation. However, many of these regulatory interactions have been explored exclusively in response to heat shock, highlighting the need for a systematic proteomics approach in which the HSF1 interactome can be simultaneously investigated under different stress conditions, including chronic protein misfolding.

To decipher new aspects of HSF1 regulation via protein partners under distinct cellular stress states, the HSF1 interactome was examined during normal growth conditions, acute heat shock, and the chronic protein misfolding stress encountered in HD. HSF1 interacts with an array of proteins with diverse cellular functions, including mRNA processing, chromatin modification, transcriptional coactivators and repressors, and DNA and RNA metabolism. Although some of these interactions are maintained under all conditions evaluated, HSF1 also interacts with a distinct network of protein partners during acute versus chronic stress. CCCTC-binding protein (CTCF) was identified as an HSF1 interactor under all three conditions. CTCF and HSF1 interact *in vivo* and directly *in vitro*; this interaction requires the DNA-binding domain (DBD) of HSF1 but does not require HSF1 to be DNA binding competent. Acute depletion of CTCF or HSF1 reveals a strong overlap of potential repression targets and, given the co-occupancy of CTCF and HSF1 at genomic loci (26), indicate that CTCF may help recruit HSF1 and facilitate HSF1-mediated regulation of gene targets. This potential cooperation between HSF1 and CTCF could reveal a novel mechanism for HSF1-mediated target gene regulation, particularly for HSF1 repression targets.

## Results

### HSF1-interacting proteins in control, heat shock, and Huntington's disease model cells

To identify proteins that interact with HSF1 in cells during normal growth conditions, in response to the acute stress of heat shock, or during the chronic stress of protein misfolding of HD, endogenous HSF1 was immunoprecipitated and interacting proteins were identified using mass spectrometry. Striatal neuron-derived cells from HD model mice possessing pathogenic polyQ-expanded Htt, STHdh^Q111/Q111^ (Q111, HD), were compared with their wildtype counterpart, STHdh^Q7/Q7^ (Q7, Control) (57). This well-characterized HD cell model recapitulates several phenotypes of HD including cellular dysfunction, aberrant localization of mHtt protein, and compromised HSF1 DNA binding and target gene expression (28, 31, 57).

**Figure 1:**
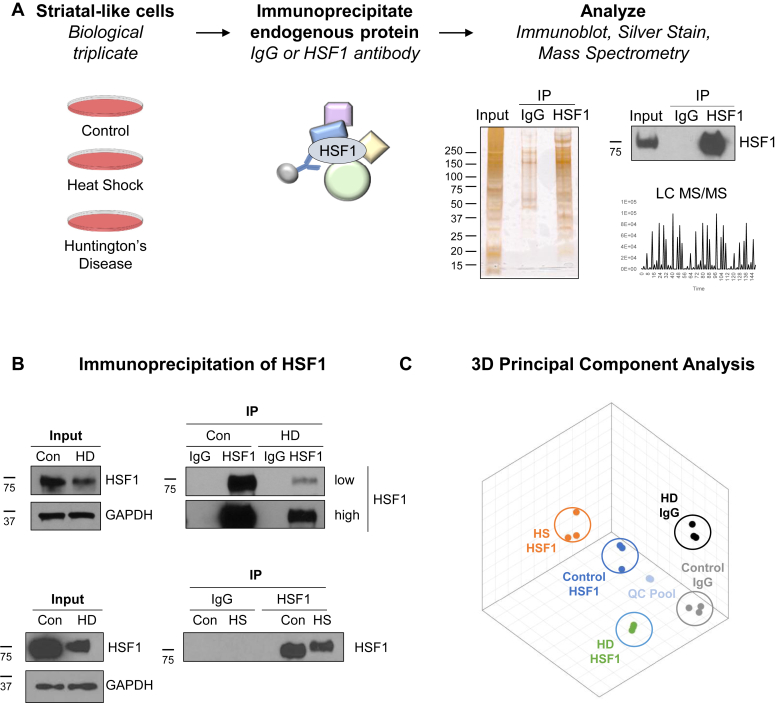
**Outline of quantitative HSF1 protein interactome approach.***A*, endogenous HSF1 immunoprecipitation from biological triplicates using polyclonal anti-HSF1 antibody covalently liked to DynaG beads. HSF1-interacting proteins were identified by immunoblotting, silver-stained SDS-PAGE gels, and quantitative LC-MS/MS to compare the magnitude of interacting proteins across conditions analyzed. IgG was used as a negative control. *B*, immunoprecipitation of endogenous HSF1 in Control (Q7), Huntington's disease (HD, Q111), or heat shock (HS) as compared with negative control IgG. *C*, 3D Principal component analyses of the proteins identified in each sample demonstrate reproducibility of triplicates in the same biological context, whereas the separation of groupings in 3D space shows the differences in the HSF1 interaction network under the different evaluated conditions. Quality control (QC) pool is an equal mixture of the 15 independent biological samples tested and was used to assess technical variability across runs.

**Figure 2:**
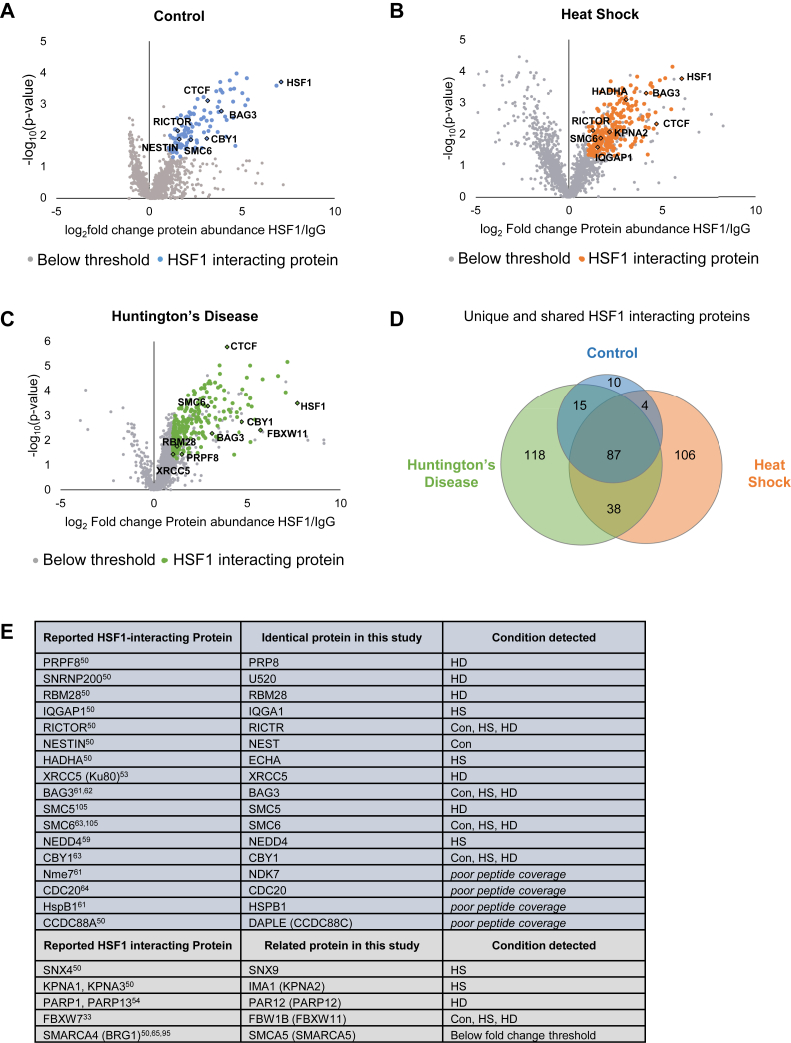
**Protein-interactors identified in control, heat shock, and Huntington's disease cell models with HSF1 immunoprecipitation–mass spectrometry.***A–C*, volcano plot of HSF1-interacting proteins in Control *A*, HS *B*, and HD *C*; proteins passing fold change >2, *p* < 0.05, and ProteinTeller probability >0.8 are highlighted in blue, orange, and green, respectively. Proteins below these thresholds are shown in gray. Several previously reported HSF1-interacting proteins and select proteins of interest are highlighted with text. *D*, Venn diagram showing the shared and distinct interactions of HSF1 observed in each condition. *E*, some of the proteins identified in this study that are identical to (*dark gray*) or highly similar to (*light gray*) previously reported HSF1-interacting proteins, with references to previous reports (33, 50, 53, 54, 59, 61, 62, 63, 64, 65, 95, 105).

To enable the identification of protein interactions with HSF1 present at physiologically relevant levels in the absence of a cross-linking agent, endogenous, native, and untagged HSF1 was immunoprecipitated and interacting proteins were identified with mass spectrometry. This is significant because HSF1 overexpression drives unnatural oligomerization and DNA binding, which could result in protein interactions that are not relevant at physiological levels of HSF1 (58). In addition, as HSF1 levels are diminished in HD owing to aberrant protein modifications and interactions, overexpression may alter the specific HSF1 interactions (33, 59). Immunoprecipitated HSF1 or negative control IgG precipitates were analyzed by ultraperformance liquid chromatography tandem-mass spectrometry (LC-MS/MS), and the HSF1 interactome was quantitatively assessed in unstressed conditions and during acute and chronic stress (Fig. 1*A*). HSF1-interacting proteins were identified in biological triplicate under the following conditions: unstressed Q7 cells (IgG immunoprecipitation [IP], HSF1 IP), Q7 cells after an acute heat shock (HSF1 IP), and HD cells (IgG IP, HSF1 IP), representing a total of 15 biological samples (5 experimental conditions × 3 biological replicas). Immunoprecipitation of HSF1 (Fig. 1, *A* and *B*, Fig. S1, *A* and *B*) demonstrates the enrichment of HSF1 and potential HSF1-interacting proteins as compared with IgG. In addition, a portion of the 15 biological samples were combined to create a QC pool used to account for any changes in the detection protocol throughout sample runs. Proteins were identified by LC-MS/MS, and runs were aligned based on the accurate mass and retention time of detected ions. In total, the initial dataset identified 12,497 unique peptides that were aligned to 1691 proteins. The coefficient of variation (% CV) for technical replicates of QC runs was 7.7%, and the biological variability was 20.9%, 29.5%, 24.2%, 18.6%, and 18.2% for the Q7 IgG, Q7 HSF1, Q7 HS HSF1, Q111 IgG, and Q111 HSF1 groups, respectively. The reproducibility of HSF1-interacting proteins identified from the three conditions was visualized with 3D principal component analysis (PCA) (Fig. 1*C*) and 2D PCA (Fig. S1*D*). Each PCA datapoint reflects the content and intensity of interacting proteins in the HSF1 or IgG IP-MS. Note that the clustering of biological replicates in 2D and 3D PCA indicates that highly similar protein components were identified in each replicate. In addition, separation of the different conditions in the PCA demonstrates the varied composition of the HSF1 interactome across conditions.

### Validation of previously identified HSF1-interacting proteins

To eliminate low-confidence HSF1 interaction partners and proteins non-specifically immunoprecipitating, several restrictions were imposed on the identified proteins. First, hit proteins in the HSF1 IP must be enriched at least twofold over levels detected in the IgG IP and this difference must be statistically significant (*p* < 0.05) as determined by a *t* test calculated on log_2_-transformed intensity values. Lastly, to ensure proteins were correctly identified with sufficient peptide coverage, hit proteins were required to have a protein teller probability of 0.8 or higher. Protein teller probability determines protein identity based on all identified peptides, penalizing single-hit proteins and assigning common peptides to the simplest number of corresponding proteins (60). Excluding HSF1 itself, of the 1691 proteins identified, only 378 (22.4%) passed these stringent requirements; hit proteins in each condition are shown in blue (Control), orange (HS), and green (HD) (Fig. 2, *A*–*C*) and comprise the high confidence protein interaction partners of HSF1 for each condition evaluated in this study. Proteins in each condition that did not pass these criteria could still represent *bona fide* HSF1 interactions and are shown in gray.

**Figure 3:**
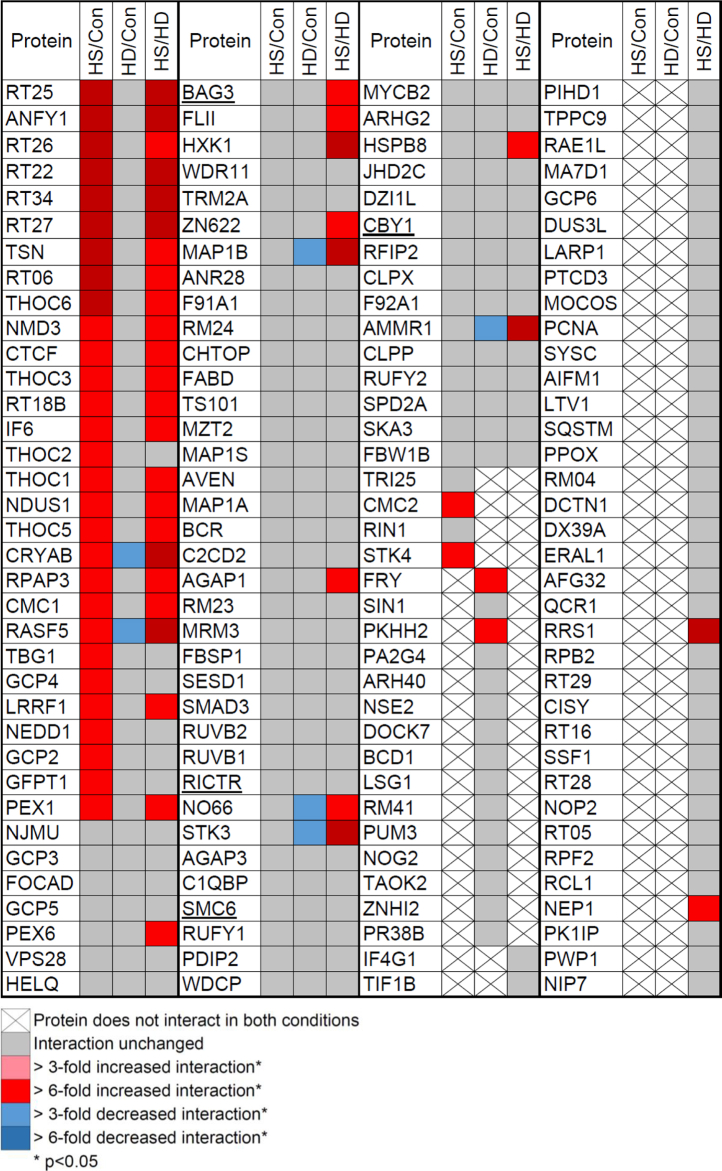
**Heat map of HSF1-interacting proteins shared in two or more conditions.** The magnitude of the interacting protein detected in each condition was compared; magnitude of interaction was calculated from robust mean normalization of raw intensity values for each protein present in the HSF1 IP (see Table S1). Interactions that changed by threefold (down, *light blue*; up, *light red*) or sixfold (up, *dark red*; down, *dark blue*) with statistical significance (*p* < 0.05) are indicated. Proteins found in similar levels across conditions are colored *gray*. Proteins indicated with crosses were not found in both indicated conditions and were not compared. Underlined proteins indicate those recapitulated from previous studies (see Fig. 2*E*).

Several previously reported HSF1-interacting proteins were detected, including BAG3 (49, 61, 62), X-ray repair cross complementing 5 (XRCC5, Ku80) (53), Pre-mRNA processing factor 8 (PRPF8) (50), rapamycin-insensitive companion of mTOR (RICTOR) (50), and structural maintenance of chromosomes protein 6 (SMC6) (Fig. 2*E* gray shading, underlined in Fig. 3 and Table S2) (63). Several HSF1-interaction partners closely functionally related to those previously described were also detected, including sorting nexin 9 (SNX9)—related to SNX4; importin subunit alpha-1 (IMA1, KPNA2)—related to KPNA3 and 4; and PAR12—related to PARP13 (HD only) (50, 54). Some of the discrepancies between the related proteins identified in this study may be attributed to differences in cell type, immunoprecipitation method, or specific stress conditions. Other identical or highly related HSF1-interacting proteins were strongly enriched in the HSF1 IP including CDC20 (64), HSPB1, and NDK7 (Fig. 2*E*) (61). However, owing to poor peptide coverage they were not above the stringent cutoff imposed for protein teller probability (Fig. 2*E*) (61).

### HSF1 protein interaction networks span a wide variety of cellular functions

The interactome of HSF1 in this neuronal-like background comprises proteins of varied cellular function. Although some proteins interact with HSF1 across multiple cellular conditions, unique interactors were also found in each condition (Table S2). The number of shared and unique HSF1-interacting proteins in control, heat shock, and HD conditions are shown in Figures 2*D* and S2, *A–C*. In general, fewer HSF1 protein interactions (10 unique) were detected that met our criteria in control conditions compared with HS (106 unique) and HD (119 unique); HD and HS shared a considerable (48%–52%) overlap, suggesting that some of these interactions may be stress dependent or common for HSF1 activation. However, HS and HD conditions also maintained many unique protein interactions, underscoring the potential differences in protein regulation exerted on HSF1 during the acute temperature stress and chronic stress of protein misfolding. In response to heat shock, HSF1 interacts with many proteins involved in nuclear import and degradation; 8% of heat shock–specific HSF1 interactions involve nuclear import machinery such as importins (α, β, 4, 5, 7, and 9) and nuclear pore proteins (Nup93), whereas 33% involve degradation, such as ubiquitin ligases (Nedd4, TRI56) and proteasomal subunits and proteases (PSA1–7, PSD11–13) (Table S1). These HS-specific interactions suggest that transient HSF1 activation involves the nuclear import machinery, interaction with RNA Pol II subunits (RPB1-3), and degradation machinery. Although these interactions are not observed in chronic stress, HSF1 does interact with many proteins specifically in HD cells (Table S2). For instance, HD-specific interactions are enriched for RNA processing factors, including many members of the DEAD-box helicase families and the RNA exosome complex. In addition, several proteins involved in DNA repair interact with HSF1 specifically in HD, including XRCC5, SMC5-6, and PARP12.

To further explore how the constellation of HSF1-interacting proteins are changed between control, heat shock, and HD conditions, the intensity of the interactions shared between two or more conditions was assessed (Fig. 3). When comparing the magnitude of HSF1-interactors, only proteins with abundance that were greater than threefold different between conditions (*p* < 0.05) were considered as high-confidence changes. With these criteria, most of the proteins interacted with HSF1 to a similar extent when comparing control and stress conditions; 78% of the shared interactions between control and HS and 92% of control and HD were preserved to a similar magnitude (gray boxes, Fig. 3). In contrast, only ∼52% of the shared interactions in HS and HD were unchanged. Interestingly, for shared interactions that were different between HS and HD conditions, all HSF1-interacting proteins interacted to a larger extent under HS conditions than in HD cells (red boxes, Fig. 3).

**Figure 4:**
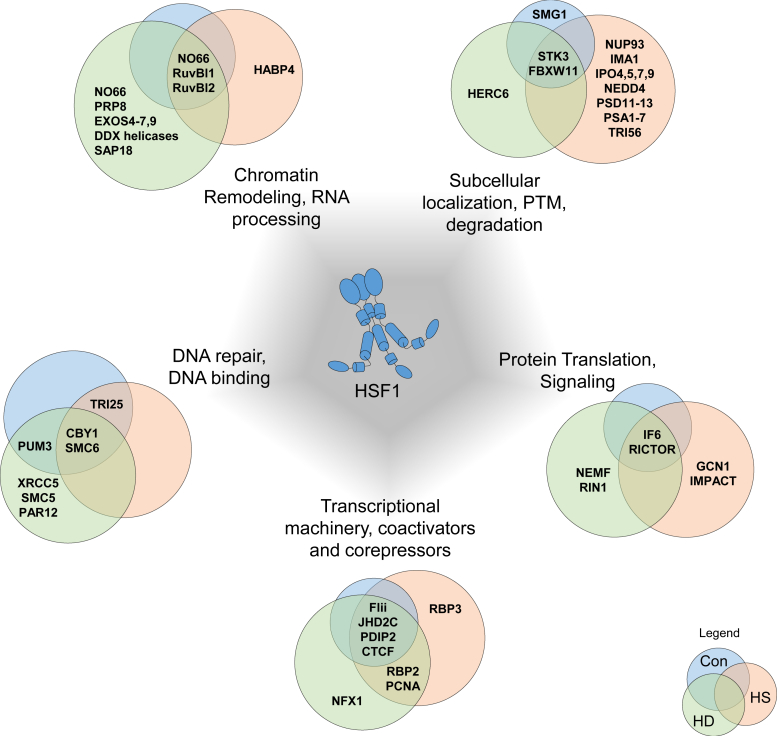
**HSF1 Interactome in the different stress conditions, showing**. HSF1-interacting proteins from select functional categories in unstressed (Con, *blue*), heat shock (HS, *orange*), and Huntington's disease (HD, *green*).

Overall, HSF1 interacts with proteins of broad cellular function including those involved in chromatin remodeling, protein trafficking, protein QC, and translation and transcriptional coactivators and repressors (Fig. 4). Of particular interest are the many chromatin remodeling proteins detected. Although this study does not differentiate between direct HSF1 interactions and those that operate as a complex containing other proteins, previous studies have demonstrated the importance of recruitment of chromatin remodelers, such as BRG1 of the SWI/SNF multiprotein chromatin remodeling complex, for HSF1-driven transcription (51, 65). Some of the chromatin remodeling proteins identified in this study include histone demethylase NO66 and RuvBl1 and RuvBl2 helicases, components of the NuA4 histone acetyltransferase complex involved in transcriptional activation. RuvBl1/2 interact with and regulate the activity of other DNA-binding proteins, such as Myc and β-catenin (66). Lastly, many different proposed transcriptional activators, such as JHD2C and canonical repressors, were found to interact with HSF1, including NFX1 and CTCF. These proteins may provide mechanistic insight into how HSF1 mediates gene repression, a largely unexplored aspect of HSF1 biology that contributes to disease conditions through a network of HSF1-repressed genes in cancer (19) and other gene targets involved in inflammation (20, 21, 22, 52).

HSF1 is well known for its role in stress-regulated gene activation, but it also serves to repress gene expression (19, 20, 21, 22, 52). Because of its role as a multi-functional transcriptional regulator, one HSF1-interacting protein of particular interest is CCCTC-binding factor (CTCF), a high-confidence interactor in unstressed, heat shocked, and HD cells (Fig. 2, *A*–*C*). CTCF is a transcriptional regulator that binds DNA and proteins, including other transcription factors, via eleven zinc fingers, to coordinate transcriptional regulation across many loci throughout the genome (67). CTCF also participates in chromatin organization, gene insulation, gene activation, and gene repression by promoting interactions of chromosomal DNA and recruiting chromatin remodeling enzymes (67, 68, 69). CTCF interacts with several other DNA-binding proteins, including Y-box–binding protein, multifunctional transcription factor YY1, and Kaiso, and regulates their function (67). In addition, a previous HSF1 chromatin immunoprecipitation (ChIP)-seq study revealed high co-occupancy of CTCF at HSF1-bound sites (26). Based on the HSF1–CTCF co-occupancy at regions with histone 4 acetylation, but lacking RNA polymerase II, it was proposed that CTCF may play a role at untranscribed regulatory elements during heat stress (26). Interestingly, CTCF also showed high co-occupancy with HSF1 at other categories of HSF1-bound loci, including promoters of HSF1 target genes (26). Because of the HSF1–CTCF interaction identified in this study, and the previous observation that CTCF and HSF1 bind at highly overlapping loci (26), we explored the functional consequences of the CTCF–HSF1 interaction.

### A direct interaction between HSF1 and CTCF is stimulated by acute stress

**Figure 5:**
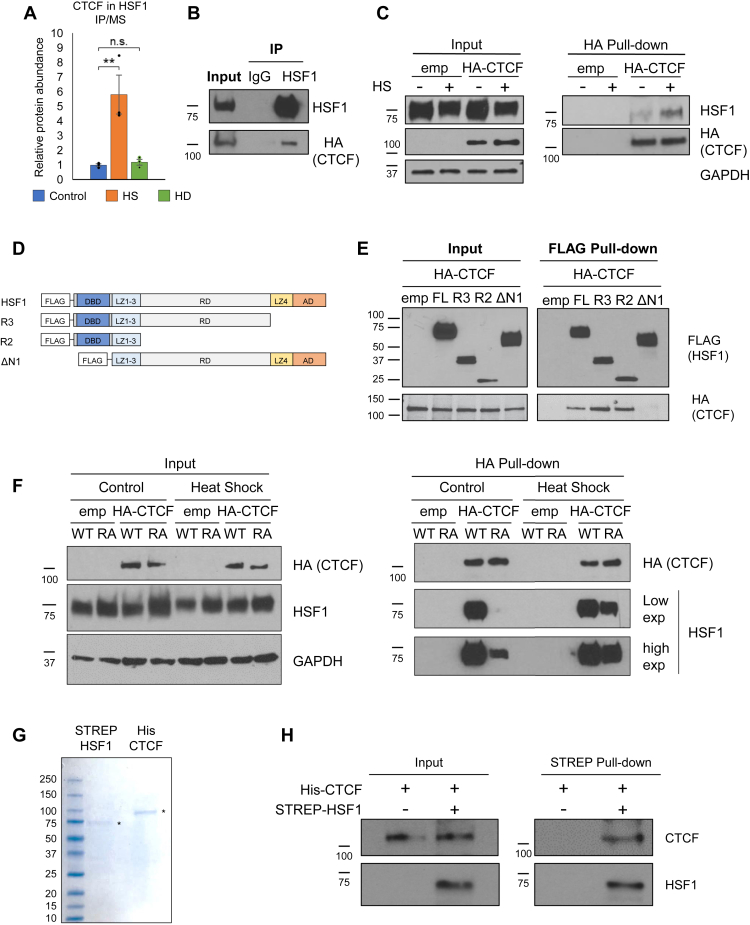
**HSF1 interacts with CTCF via the DBD but does not require HSF1 DNA binding.***A*, HSF1 IP-MS analysis demonstrated increased CTCF levels in the HSF1 IP; individual datapoints for biological replicates are shown as *black dots*, whereas the bars in each condition indicate the average protein abundance relative to control conditions. *B*, endogenous HSF1 or IgG control immunoprecipitation from HEK 293T cells transfected with a plasmid expressing HA-CTCF. *C*, HA pull-down from HEK 293T cells transfected with HA-CTCF in control or heat shock (30 min, 42 °C) conditions. *D*, diagram of FLAG-tagged HSF1 truncations used in *E* with the FLAG epitope tag and HSF1 domains indicated. *E*, stable HSF1-knockdown cells transfected with a plasmid expressing HA-CTCF and either empty vector or FLAG-HSF1 wildtype or the indicated truncated proteins; the FLAG-HSF1 pull-down was conducted and followed by immunoblotting with anti-HA (CTCF) antibody. *F*, stable HSF1-knockdown cells transfected with empty vector or plasmids expressing HA-CTCF and WT HSF1 or R71A HSF1, harboring a mutation that abrogates HSF1 DNA binding, followed by HA pull-down and immunoblotting with anti-HSF1 antibody. *G*, Coomassie-stained SDS-PAGE gel of purified STREP-HSF1 and His-CTCF used in *H*. *H*, *in vitro* STREP purification of His-CTCF alone or together with STREP-HSF1, followed by immunoblotting with anti-CTCF and anti-HSF1 antibodies.

To independently validate the quantitative CTCF–HSF1 interaction observed from HSF1 IP-MS (Fig. 5*A*), endogenous HSF1 was immunoprecipitated from HEK 293T cells transfected with hemagglutinin-tagged CTCF (HA-CTCF) and HA-CTCF was probed by immunoblotting. HSF1 robustly interacted with CTCF in unstressed cells but did not interact with the negative control IgG precipitate (Fig. 5*B*). In addition, the increased HSF1–CTCF interaction observed in heat-stressed cells by IP-MS (Fig. 5*A*) was recapitulated by pull-down of HA-CTCF in control and heat shock–treated HEK 293T cells, with which HSF1 co-purified to a greater magnitude in heat shock conditions (Fig. 5*C*).

HSF1 harbors multiple domains of distinct function. To determine which region of HSF1 interacts with CTCF, plasmids encoding FLAG-tagged HSF1 truncations were constructed (Fig. 5*D*) and expressed by transfection into 293T stable HSF1-knockdown cells (Fig. S3*A*) to prevent hetero-multimerization with endogenous HSF1. CTCF co-purified only with HSF1 truncations containing the DBD, whereas HSF1 ΔN1, lacking the DBD, failed to interact with CTCF (Fig. 5*E*). To evaluate whether the interaction with CTCF requires HSF1 that is competent for DNA binding, the critical Arg residue (R) of the HSF1 “SFVRQ” DNA binding recognition helix was mutated to Ala (HSF1 R71A). This Arg residue is necessary for HSF1 DNA binding competence and makes sequence-specific contacts to the guanine of the nGAAn heat shock element (HSE) sequence, to which HSF1 binds (70, 71, 72, 73). In stable HSF1 knockdown HEK 293T cells, both wildtype HSF1 and HSF1 R71A interacted with CTCF under control and heat shock conditions (Fig. 5*F*). Similar results were obtained by comparing HA-CTCF co-purification with WT and R71A HSF1 in HSF1^−/−^ mouse embryonic fibroblasts (Fig. S3*B*). These results indicate that the DNA binding activity of HSF1 is not required for interaction with CTCF.

To determine if the HSF1–CTCF interaction requires additional proteins *in vivo*, an *in vitro* pull-down using purified components was conducted. Human, codon optimized STREP-HSF1 and CTCF-His were expressed in *Escherichia coli* and purified by affinity chromatography (Fig. 5*E*). In an *in vitro* STREP pull-down, we observed co-purification of CTCF in the presence of STREP-HSF1, but not in the absence of HSF1 (Fig. 5*H*). This indicates that CTCF and HSF1 interact directly *in vitro* in the absence of other mammalian proteins.

### CTCF and HSF1 show overlapping profiles of potential repression targets

**Figure 6:**
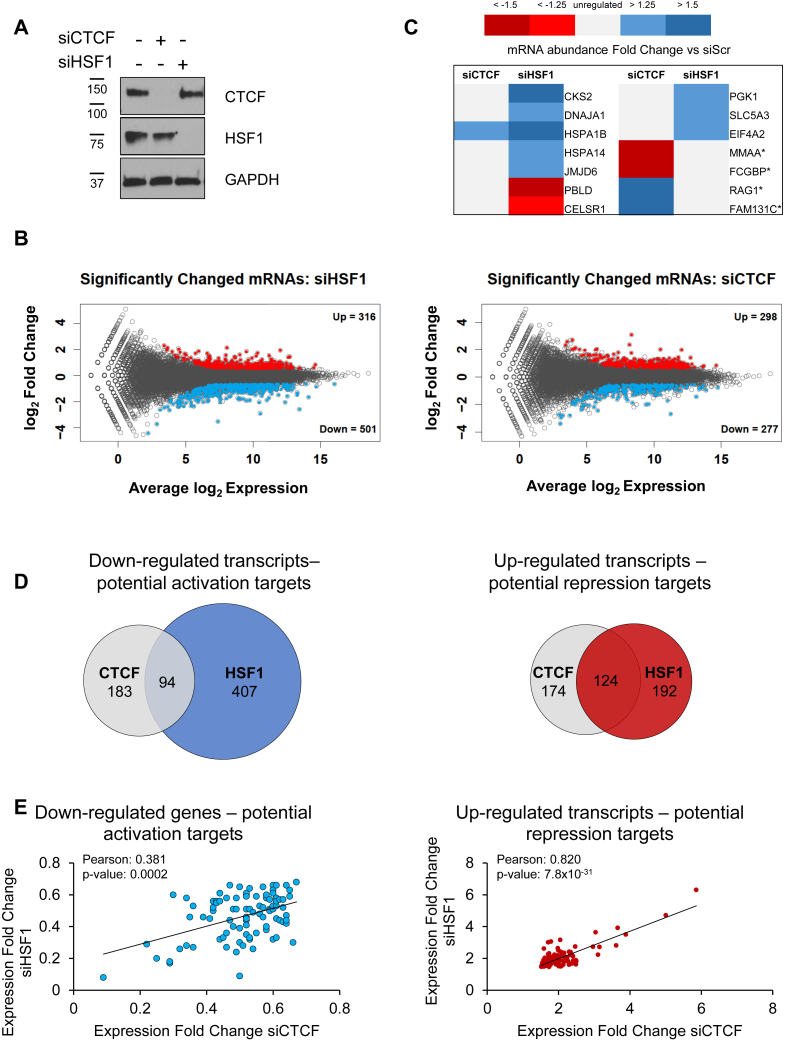
**Role of CTCF in HSF1-mediated basal transcription.***A*, protein extracts generated from HEK 293T cells transfected for 48 h with siRNA targeting HSF1, CTCF, or scrambled control. *B*, DESeq2 analysis revealed significant (*p* < 0.001) high confidence changes in transcript abundance in the HSF1 or CTCF siRNA treated cells. *C*, transcript abundance of previous established HSF1 (19, 106) or CTCF targets (69) (∗ indicates CTCF target genes) as assessed by RNA sequencing (n = 4). *D*, overlap of down- and up-regulated genes for siHSF1 or siCTCF treated cells; numbers indicate the quantity of distinct transcripts in each category. *E*, Pearson correlation of down- and up-regulated genes for siHSF1- and siCTCF-treated cells.

Although several studies have explored stress-induced gene transcription by HSF1, fewer have focused on how HSF1 exerts control over basal transcription and what regulatory factors may affect this. As CTCF may likely exert its regulatory function at the level of chromatin architecture and the three-dimensional chromatin structure is largely conserved in response to heat shock (74), this study focuses on HSF1 and CTCF in the unstressed state. To begin to understand how CTCF may influence basal HSF1-mediated transcription, transcript abundance was analyzed by RNA-seq in HEK 293T cells during transient siRNA-mediated knockdown of CTCF or HSF1. This study explored transcriptional changes during normal growth conditions, during which the CTCF–HSF1 interaction is observed (Fig. 5). Four biological replicates of each condition were collected and demonstrated strong correlation of transcriptional profiles with Spearman coefficients greater than 0.95 (Fig. S4*A*). CTCF and HSF1 knockdowns successfully and robustly depleted target transcripts (14% and 23% of HSF1 and CTCF mRNA abundance, respectively, were observed compared to with negative control siScr) and do not impact partner protein, transcript abundance, or splicing (Fig. 6*A*, Fig. S4, *B* and *C*). Transcriptional changes of siRNA-targeted and negative control samples were assessed with DESeq2, categorizing genes as significantly changed as either high or low confidence (*p* < 0.001), unregulated, or unexpressed. Genes with a minimum fold change of 1.25 were considered high confidence calls and are reported here as up- or down-regulated. Under these conditions, 501 down-regulated transcripts were observed upon HSF1 depletion; these could be basal targets for HSF1 activation, although many are likely regulated in a non–HSF1-dependent manner (Fig. 6*B*). Similarly, 316 transcripts were found with higher abundance in siHSF1-treated cells, some of which may be HSF1 repression targets (Fig. 6*B*).

Changes in a battery of established CTCF and HSF1 gene targets demonstrate that knockdown in these conditions resulted in predicted changes in target gene expression. Transcript levels of established HSF1 targets were altered, including both activation and repression targets. For example, the abundance of HSF1 activation targets DNAJA1, HSPA1B, HSPA14, JMJD6, and CKS2 was reduced in siHSF1 treatment (Fig. 6*C*). HSF1 repression targets PBLD and CELSR1 were de-repressed in siHSF1-treated cells (Fig. 6*C*). Expression of the MMAA and FCGBP genes, established CTCF repression targets, is de-repressed in siCTCF-treated cells (69) (Fig. 6*C*). On the other hand, activation targets of CTCF such as RAG1 and FAM131C showed lower mRNA abundance in siCTCF-treated cells (Fig. 6*C*). Comparison of the up- and down-regulated genes in siHSF1 or siCTCF cells revealed a strong overlap, particularly for genes with potential HSF1 repression. Although 94 of the 501 down-regulated genes in siHSF1-treated cells were shared with siCTCF-treated cells, 124 of the 316 up-regulated genes in siHSF1 cells were up-regulated also in the absence of CTCF (Fig. 6*D*). To assess if the individual genes shared in siHSF1 and siCTCF treatments were changed to a similar magnitude, the transcriptional changes of potential activation and repression targets were compared. The Pearson correlation of potential repression targets was higher than all detected transcripts (Fig. S4*D*) and more correlated than potential activation targets, showing a Pearson correlation of 0.820 versus 0.381 (Fig. 6*E*). The strongly correlating changes in mRNA levels in siHSF1 and siCTCF cells likely reflects the importance of CTCF in preparing chromatin architecture for transcriptional regulation. In addition, the physical interaction of HSF1 and CTCF under non-stress and stress conditions demonstrates the importance of controlling HSF1 and its interactome in both physiological and pathophysiological conditions.

## Discussion

The regulation of HSF1 in response to acute stress has been studied since the discovery of the heat shock response in the early 1960s (75). More recently, the dysregulation of HSF1 and the crucial roles this stress-protective transcription factor plays in the pathophysiological contexts of both neurodegenerative disease and cancer have been of great interest (18, 19, 33). Studies have shown the diminution in HSF1 levels in neurodegenerative diseases including HD, Alzheimer's disease, and Parkinson's disease and the dysregulation of HSF1 target gene expression (33, 59, 76, 77, 78). In HD, HSF1 genome-wide binding and transcriptional network regulated by HSF1 are disrupted and impaired HSF1 function further exacerbates chronic protein misfolding (28, 33, 79). In contrast, in cancer, high levels of active, nuclear HSF1 support malignancy by coordinating a transcriptional signature partially distinct from heat shock, including targets that regulate cell cycle progression, chaperone production, and repression of apoptotic factors (19). A more thorough understanding of the regulation of HSF1 could offer new insights into how HSF1 is altered in different disease states. Because one major mechanism for HSF1 regulation is driven by protein–protein interactions (22, 24, 50, 51, 54), this study explores how the networks of HSF1-interacting proteins are altered in control conditions and those of heat shock and chronic protein misfolding such as that observed in HD. In addition, in contrast to previous HSF1-proteomics studies, this study utilized mouse cells derived from the striatum of the brain, the area most affected in HD, and therefore may reflect regulatory interactions specific to cells in the central nervous system in a normal context and in disease.

This study found a highly diverse group of HSF1-interacting proteins with broad cellular functions. Overall, HSF1 interacts with more proteins in response to acute or chronic stress compared with basal conditions, where many of these stress-induced interactions are shared between heat shock and HD but are of lower magnitude in HD. One such protein is serine threonine kinase (STK3), which is activated by pro-apoptotic stimuli; STK3 showed an ∼ sixfold reduced interaction in HD compared with control cells and interacted with HSF1 ∼ tenfold more in acute heat shock than HD; HSF1 is known to be regulated by various post-translational modifications, including phosphorylation events, and the role of STK3 in modulating HSF1 is currently unexplored. In addition, chibby family member 1 (CBY1) was identified in our study in all conditions tested and in a previous unbiased, multi-protein interactome study (63); CBY1 is known to interact with and regulate several other proteins, including β-catenin and thyroid cancer-1 (TC-1) (80). Further investigation of HD-specific interactions may reveal new ways that HSF1 regulation is altered in chronic protein misfolding stress. Interestingly, HSF1 in HD was found to interact with several proteins involved in DNA repair including SMC5 and SMC6, XRCC5, and PARP12. DNA repair pathways have been implicated in the advancement and onset of HD and other trinucleotide repeat diseases as they can contribute to the further expansion of the disease-causing trinucleotide tract (81, 82). Investigation of these protein interactions may reveal a role for HSF1 in genome integrity in HD as previously shown for mammary tumors (54). Another protein of interest identified in HD conditions in this study is nuclear export mediator factor (NEMF), a component of the ribosome QC complex. In yeast, translational stress activates HSF1 in a manner distinct from heat shock, and the highly conserved yeast homolog of NEMF, Tae2, is critical for the transmission of translational stress to yeast HSF (83). Data described here suggest that the HSF1–NEMF interaction, and the potential role this interaction serves in the RQC-stress response pathway, may be conserved in higher eukaryotes. In addition, the possible functional consequence of the interaction of HSF1 with pre-mRNA processing factors observed by us (PRP8, PRP17), and PRP8 by others (50), has yet to be explored.

Although many chaperone proteins including Hsp70, Hsp90, and subunits of the TRiC–CCT complex were detected in the HSF1 immunoprecipitation, most were not sufficiently enriched in the HSF1 IP over the negative control IgG IP to meet our strict threshold. This may, in part, be due to the low affinity of the HSF1-chaperone interactions described. For instance, a recent study found that HSF1 only interacts with a subset of Hsp90 conformations and thus the overall affinity of HSF1 for endogenous HSP90, which is sampling many different conformations, is relatively weak (84). A similarly low-affinity interaction of yeast HSF1 with the CE2 region of Hsp70 was biochemically characterized (10). Furthermore, multiple Hsp70-binding sites have different affinities for human HSF1, the interaction of which functions to unwind HSF1 oligomers (85). Lastly, the profound technical differences in HSF1 IP-MS techniques used in this study as compared with those studies optimized for the identification of chaperone interacting proteins (86) could certainly contribute to the relatively small number of HSF1-interacting chaperone proteins identified in this study.

It is currently unclear why and how HSF1 interacts with more proteins in response to stress, but this could be a function of its oligomerization, nuclear localization, or stress-induced post-translational modifications that drive specific regulatory interactions. For example, in the case of CTCF, a nuclear protein, the retention of HSF1 in the nucleus and increased level of chromatin-bound HSF1 would explain the increased interaction in response to acute heat stress as compared with unstressed cells. Whether these other, unexplored HSF1 interactors alter HSF1 function remains to be determined. What is clear is the distinct HSF1-interacting networks in unstressed, heat shock, or HD cells; future studies of these protein interactors may reveal new facets of HSF1 biology or new HSF1 regulatory mechanisms. In particular, determining if any of the observed interactions are striatum specific may provide insight into how HSF1 is regulated in the striatum, the area of the brain that is most affected in HD (35).

One highly interesting HSF1-interacting protein observed was CTCF, which is primarily known for its role in chromatin organization and transcriptional repression (68, 87). CTCF and HSF1 interact *in vivo* and directly *in vitro*, and the potential repression targets of CTCF and HSF1 overlap more strongly than activation targets. A previous study found a high occupancy of CTCF at HSF1-bound, particularly at untranscribed regions (26). A more recent study found that, among known and other proposed coregulators, CTCF is detected at HSE-bound HSF1 but not at mutated HSEs (88). Based on these innovative studies, the direct protein interaction observed in our study, and the strong correlation of upregulated genes in HSF1 or CTCF knockdown, we suggest that CTCF may be important for targeting HSF1 to the CTCF-rich regions in the chromatin and in HSF1-mediated gene repression.

CTCF could affect HSF1-mediated transcription in several ways, including establishing and altering chromatin looping interactions between transcription regulatory elements such as promoters, enhancers, and insulators. Altered chromatin interaction probabilities could, in turn, affect RNA Pol II regulation at connected distal regions likely via other mechanisms. It remains to be determined whether CTCF can recruit HSF1 to CTCF-primed regions accessible for transcription factor binding, or by HSF1 recruiting CTCF at HSE-bound sites (26, 67, 88, 89, 90, 91). CTCF can mediate a versatility of changes in chromatin architecture by preventing the encroachment of heterochromatin and by recruiting chromatin remodeling proteins, including enzymes catalyzing histone acetylation or methylation (67, 68, 92, 93). Like many transcription factors, HSF1–DNA binding depends on the chromatin architecture; in some cases, protein interaction partners of HSF1 influence these chromatin changes (25, 94). HSF1 interacts with replication protein A, and this complex recruits histone chaperone FACT, resulting in opening of the chromatin structure (50). In addition, the transcriptional activation domain of HSF1 interacts with BRG1, a SWI/SNF chromatin remodeling complex, and this is important for the production of full-length Hsp70 (65, 95).

Although this study does not encompass how CTCF may affect genome-wide binding of HSF1, it raises questions of the purpose of the HSF1–CTCF interaction. Whether CTCF is involved in facilitating HSF1 DNA binding through protein-protein interactions, or via chromatin remodeling, remains to be determined. Interestingly, CTCF has been implicated in regulation of DNA binding of other proteins, including TAF3 (96), which was previously shown to be necessary for chromatin architecture and HSF binding in *Drosophila* cells (97). Future studies will determine whether CTCF modulates HSF1 target gene transcription, but our data suggest that the involvement of CTCF may be, in part, for HSF1 basal repression targets. Future studies may also address how HSF1 stress-induced transcription is affected by CTCF and what other factors affect this protein–protein interaction, like post-translational modifications. The repressive function of HSF1 has become increasingly important in recent light of the targets repressed by HSF1 that contribute to disease, including inflammation, apoptosis, and protein misfolding diseases, such as Tau (19, 20, 21, 22, 52, 98, 99, 100).

## Experimental Procedures

### Cell culture and cell lines

Cell lines used in this study were as follows: mouse-derived striatal cells STHdh(Q7) and STHdh(Q111) (Coriell Cell Repositories), 293T human embryonic kidney (HEK) cells (ATCC CRL-1573), stable shScr and shHSF1 HEK 293T cells (generated in this study), and HSF1^−/−^ mouse embryonic fibroblast (MEF) cells (from Dr Ivor Benjamin, Medical College of Wisconsin). Striatal cells were maintained at 33 °C in Dulbecco's modified Eagle's medium (DMEM) supplemented with 10% fetal bovine serum (FBS) and 91 μg mL^−1^ Normocin (InvivoGen). HEK 293T cells were grown at 37 °C in DMEM supplemented with 10% FBS and 91 μg mL^−1^ Normocin. HSF1^−/−^ MEF cells were grown at 37 °C in DMEM supplemented with 10% FBS, 0.1 mM nonessential amino acids, 100 U ml^−1^ penicillin/streptomycin, and 55 μM β-mercaptoethanol. Striatal cells (STHdhQ7 and STHdhQ111) were authenticated by immunoblotting with mHtt–specific antibody (MAB2166) (33). The HSF1^−/−^ MEF cells were authenticated by immunoblotting for HSF1 with multiple anti-HSF1 antibodies (Enzo 10H8, and Bethyl [5]).

### Heat shock

Cells treated with heat shock were incubated at 42 °C for 30 min and compared with cells grown at normal growth temperatures (33 °C for StHdh cells, 37 °C for 293T and MEF cells).

### Endogenous HSF1 immunoprecipitation

For HSF1 IP-MS analysis, endogenous HSF1 was immunoprecipitated from cells lysed in IP buffer (20 mM HEPES, 5 mM MgCl_2_, 1 mM EDTA, 100 mM KCl, 0.03% NP-40, 1% Triton X-100) supplemented with 1X HALT protease and phosphatase inhibitor cocktails (ThermoFisher). Protein lysates were cleared with centrifugation (14,000 r.c.f., 4 °C, 15 min), and protein concentrations were quantified with bicinchoninic acid assay method (Pierce). Samples were normalized to 1 mg ml^−1^ and precleared with DynaG beads for 3 h. DynaG magnetic beads covalently cross-linked to either negative control IgG (Bethyl) or HSF1 (Bethyl) (5) were added to samples and incubated overnight at 4 °C rotating. Beads were washed four times with IP buffer and eluted with 50 μl of TES (10 mM Tris pH 7.5, 1 mM EDTA, 0.5% (w/v) SDS) at 65 °C, 10 min. Eluates were snap frozen in liquid nitrogen until being processed by mass spectrometry, immunoblot, or silver-stained gel analysis. For HSF1 immunoprecipitation not used for mass spectrometry, the protocol above was identical except DynaG beads were not covalently linked to antibodies. Instead, antibodies were incubated with lysates overnight, and subsequently, beads were added and incubated for 3 h before the washes and elution. HA and FLAG pull-downs were performed in the same manner using anti-HA magnetic beads (Thermo Scientific) or anti-FLAG M2 magnetic beads (Sigma). HA pull-downs were eluted in 0.1 M glycine pH 2.0, and samples were neutralized using 1M Tris pH 8.5 or TES; FLAG pull-downs were eluted in TES.

### Covalent linkage of antibody to beads

To couple the beads and antibody, DynaG beads were incubated with IgG or HSF1 in phosphate buffered saline (PBS) for 3 h at 4 °C (30 μl beads per 20 μg antibody, 1 ml PBS). Antibody-coupled beads were washed three times with 0.2 M sodium borate pH 9.0 and cross-linked with freshly made 20 mM dimethylpimelimidate dissolved in 0.2 mM sodium borate pH 9.0. Cross-linking was performed for 40 min at room temperature with rocking, followed by three washes with acid wash buffer (0.58% v/v acetic acid, 150 mM NaCl) and three washes with ice cold PBS. Beads were prepared and used the same day for subsequent immunoprecipitation experiments.

### Mass spectrometry

Samples were diluted in TES and 1X Laemmli/SDS buffer, which were supplemented with 1 μl 200 mM DTT each, and heated at 70 °C for 10 min. Forty-nine microliters of each sample was loaded onto an Invitrogen NuPAGE 4% to 12% SDS-PAGE gel (for 1D protein separation) and run for approximately 5 min to electrophorese all proteins into the gel matrix. The entire molecular weight range was then excised in a single gel band and subjected to standardized in-gel reduction, alkylation, and tryptic digestion. Following lyophilization of the extracted peptide mixtures, samples were resuspended in 12 μl of 2% acetonitrile/1% TFA supplemented with 12.5 fmol μl^−1^ yeast ADH. From each sample, 3 μl was removed to create a QC pool sample.

Quantitative LC-MS/MS was performed using 4 μl of each sample, using a nanoACQUITY UPLC system (Waters Corp) coupled to a Thermo Q Exactive HF high resolution, accurate mass tandem mass spectrometer (Thermo) via a nanoelectrospray ionization source. Briefly, the sample was first trapped on a Symmetry C18 20 mm × 180 μm trapping column (5 μl min^−1^ at 99.9/0.1 v/v water/acetonitrile), after which the analytical separation was performed using a 1.8 μm ACQUITY HSS T3 C18 75 μm × 250 mm column (Waters Corp) with a 90-min linear gradient of 5% to 30% acetonitrile with 0.1% formic acid at a flow rate of 400 nl min^−1^ with a column temperature of 55 °C. Data collection on the Q Exactive HF mass spectrometer was performed in a data-dependent acquisition mode with a r = 120,000 (at m/z 200) full MS scan from m/z 375 to 1600 with a target automatic gain control value of 3 × 10^6^ ions followed by 15 MS/MS scans at r = 30,000 (at m/z 200) at a target automatic gain control value of 5 × 10^4^ ions and 45 ms. A 20-s dynamic exclusion was employed to increase depth of coverage. The total analysis cycle time for each sample injection was approximately 2 h.

Following 19 total ultraperformance liquid chromatography–MS/MS analyses (excluding conditioning runs, but including four replicate QC injections), data was imported into Rosetta Elucidator v 4.0 (Rosetta Biosoftware, Inc), and analyses were aligned based on the accurate mass and retention time of detected ions (“features”) using PeakTeller algorithm in Elucidator. Relative peptide abundance was calculated based on area under the curve of the selected ion chromatograms of the aligned features across all runs. The MS/MS data were searched against the SwissProt *Mus musculus* database (downloaded in May 2017) with additional proteins, including yeast ADH1, bovine serum albumin, as well as an equal number of reversed-sequence “decoys” for false discovery rate determination. Mascot Distiller and Mascot Server (version 2.5, Matrix Sciences) were utilized to produce fragment ion spectra and to perform the database searches. Database search parameters included fixed modification on Cys (carbamidomethyl) and variable modifications on Meth (oxidation) and Asn and Gln (deamidation). After individual peptide scoring using the PeptideProphet algorithm in Elucidator, the data were annotated at a 1% peptide false discovery rate.

Four microliters of peptide digest (∼30% of each sample) were analyzed by ultraperformance liquid chromatography-tandem mass spectrometry (LC-MS/MS). A QC pool containing an equal mixture of each sample was analyzed first, after every fifth sample, and end of the sample set (four times total). Individual samples were analyzed in a random order. Next, data were imported into Rosetta Elucidator v 4.0 and all LC-MS/MS runs were aligned based on the accurate mass and retention time of detected ions (“features”), which contained MS/MS spectra using PeakTeller algorithm and intensity-scaled based on a robust mean (10%) normalization of the identified features. The overall dataset had 495,664 quantified isotope (peptide) groups. In addition, 330,924 MS/MS spectra were acquired for peptide sequencing by database searching (see Experimental Procedures). Following database searching and peptide scoring using the PeptideProphet algorithm, the data were annotated at a 1% peptide false discovery rate, resulting in identification of 12,497 peptides and 1691 proteins. All proteins were intensity-scaled to levels of HSF1 and subsequent analyses were from these normalized protein levels.

### Identification of hit proteins

To remove non-specific proteins or proteins with poor peptide coverage from consideration, only proteins with a twofold or higher abundance in the HSF1 over the IgG IP (*p* < 0.05) and a ProteinTeller probability of ≥0.80 were used in subsequent analysis. Q7 IgG was used as a negative control for Q7 control HSF1 (unstressed) and Q7 HS HSF1 (heat shock); Q111 IgG was used for Q111 HD. *t* tests were calculated on log_2_-transformed data for each of these comparisons using a two-tailed heteroscedastic *t* test in Excel (Microsoft). Of the 1691 proteins identified, 378 (22.4%) passed the aforementioned cutoff requirements.

### Generation of 293T HSF1 stable knockdown cells

Stable HSF1 knockdown cells were generated following the second-generation Addgene protocol. Briefly, 293T cells were transfected with psPAX2 (viral packaging), VSVG (viral envelope) plasmids, and either non-targeting sequence shScr plasmid (pLKO.1) or shHSF1 plasmid (TRCN0000007480, Sigma). Transfection was completed with Lipofectamine LTX Reagent (ThermoFisher) following protocol instructions using 21 μg PAX2, 7 μg VSVG, and 28 μg Sigma Mission TRC1 lentiviral shRNA plasmids. Approximately 18 h post transfection, the media was replaced with fresh media. Approximately 48 h post transfection, viral particles were harvested. Supernatant containing lentiviral particles was filtered with a 0.45-μm PES filter to remove any cells from viral production.

Virus-containing, cleared supernatant was applied to 293T cells in standard grown conditions. Twenty-four hours after viral particle addition, media was changed to DMEM containing 0.25 μg/ml puromycin for selection. This concentration of puromycin was determined to be the lowest concentration at which 100% of parental HEK 293T cells would die after 3 days of treatment. After 2 weeks of puromycin selection, no more cell death was observed. shScr and shHSF1 stable knockdowns were analyzed for loss of HSF1 protein to assess knockdown.

### Transfection of plasmid DNA and transient siRNA knockdown

Plasmid transfections in 293T cells were completed using Lipofectamine LTX (Invitrogen) according to the manufacturer's protocol (MAN0007822) and were incubated for 24 h before immunoprecipitation experiments. Plasmid transfections in HSF1^−/−^ MEFs with electroporation used the SE Cell Line 4D-Nucleofector (Lonza) with 2 × 10^6^ cells per transfection and 2 μg plasmid DNA according to the manufacture's protocol. Cells were used for immunoprecipitation experiments 24 h after transfection.

Silencing RNAs (siRNA) were obtained from Thermo Fisher Scientific (Silencer Select, assay ID s6950 and s20966 for human HSF1 and CTCF, respectively) and Qiagen (non-targeting siRNA) for negative control scramble (1022076). Transient siRNA knockdowns were completed with Lipofectamine RNAiMAX according to the manufacture's protocol using 25 pmol of siRNA per well of a 6-well plate. The duration of transient knockdown was 48 h before analysis of transcript abundance with qRT-PCR, DNA binding with chromatin immunoprecipitation-qPCR, or immunoblot analysis.

### RNA extraction, sequencing, and analysis

RNA was extracted and purified from mammalian cells after 48 h of silencing (four biological replicates for each condition) using RNEasy extraction kit (Qiagen) according to the manufacturer's instructions using QIAshredder columns for homogenization. Library preparation and Illumina sequencing were completed by GeneWiz using the Total RNA process with polyadenylation selection using 2 μg of RNA as input.

Quality of sequencing was assessed with FASTQC and showed samples had sequencing depth of 26 to 36 M reads per sample. Reads were filtered for quality and mapped to the human genome (hg19) using TopHat2 (101). Significant changes in mRNA levels between siRNA treatments were called with DESeq2 (102) with a threshold of *p* > 0.001. Significantly changed genes were broken into high confidence (HC) changes and low confidence (LC) changes using fold change 1.25 as a cutoff. Fold change of HC genes were increased (UpHC) or decreased (DownHC) by 1.25 or more with a *p* value < 0.001. Fold changes of LC genes were increased (UpLC) or decreased (DownLC) by less than 1.25 with *p* < 0.001. Genes with a low read count (<0.1 normalized reads in all conditions) were categorized as UnExp. Genes with a detectable mRNA expression (minimum of 0.1 normalized reads in any condition) but no significant changes detected with DESeq2 were categorized as unregulated (UnReg). Downstream analysis was conducted only for transcripts with a minimum normalized read count of 10 per gene. Browser images were generated using Integrated Genomics Viewer (103) with density normalized mRNA counts. The raw FASTQ files and density normalized BigWig files can be accessed via Gene Expression Omnibus (GEO; https://www.ncbi.nlm.nih.gov/geo/) using the access code GSE155541.

### Protein purification of HSF1 and CTCF

Plasmids were constructed containing STREP-tagged HSF1 or His_6_-tagged CTCF (pET-15b, Addgene) and were codon-optimized for bacterial expression. Plasmids were transformed into BL21 (DE3) *E. coli*. Overnight cultures originating from a single colony were diluted 1:100 and grown to log phase at an OD_600_ of ∼0.5 at which point protein expression was induced by addition of 1 mM isopropyl 1-thio-β-D-galactopyranoside overnight at 15 °C. Cells were harvested by centrifugation (10,000 r.c.f., 10 min, 4 °C). For STREP-tagged purification, bacterial cell pellets were lysed with Buffer NP (50 mM NaH_2_PO_4_, 300 mM NaCl, pH 8.0) supplemented with HALT protease inhibitors (Thermo Fisher). Cells were lysed with sonication on ice (20-s bursts, 80 s total processing time), and lysates were cleared with centrifugation (20,000 r.c.f., 15 min, 4 °C) and applied to a StrepTrap column (GE Heath Sciences) using an Akta Pure FPLC (GE Health Sciences) at a flow rate of 0.25 ml min^−1^, washed with six column volumes of Buffer NP until the A_280_ was flat, and bound proteins were eluted with Buffer NP supplemented with 2.5 mM desthiobiotin. Fractions containing STREP-HSF1 were analyzed with SDS-PAGE and buffer exchanged using an Amicon Ultra Centricon (molecular weight cut-off of 10,000 Da) into buffer containing 25 mM HEPES pH 7.5 and 150 mM NaCl. For His-tagged purification, cells were resuspended in NiNTA Lysis Buffer (50 mM HEPES pH 7.5, 300 mM NaCl, 20 mM imidazole HCl) and homogenized with three passages through French pressure cell at >12,000 p.s.i. Cell lysates were cleared with centrifugation as above and incubated with 2 ml of bed volume of Ni-NTA Agarose (Qiagen) for 2 h, 4 °C, rocking. Beads were washed four times with NiNTA Lysis Buffer supplemented with an additional 20 mM imidazole and bound proteins eluted with NiNA Elution Buffer (50 mM HEPES pH 7.5, 300 mM NaCl, 250 mM Imidazole HCl). Fractions were analyzed with SDS-PAGE and immunoblot. His-CTCF was buffer exchanged using an Amicon Ultra Centricon (molecular weight cut-off of 10,000 Da) into buffer containing 25 mM HEPES pH 7.5, 150 mM NaCl, and 1 μM ZnCl_2_ (104).

### HSF1–CTCF *in vitro* binding assay

Binding assays were performed in 1 ml of Buffer NP (50 mM NaH_2_PO_4_, 300 mM NaCl, pH 8.0) with 5 nM of purified His-CTCF alone or STREP-HSF1 and His-CTCF together. Proteins were incubated for 1 h at 4 °C with rocking and 2 μl of MagStrep type 3 XT resin (IBA) were added and incubated for 30 min at 4 °C with rocking. Beads were washed five times with Buffer NP; interacting proteins were eluted with 25 mM desthiobiotin and analyzed with immunoblot.

### Immunoblot analysis

Protein extracts were electrophoresed on 4% to 20% SDS-PAGE and transferred to nitrocellulose membranes (Bio-Rad 0.2 μm) using Transblot Turbo (Bio-Rad) in Tris–glycine buffer (25 nM Tris base, 200 mM glycine) at 25 V. Immunoblots were blocked for 1 h at room temperature with 5% (w/v) milk in PBS supplemented with 0.25% Tween 20 (PBST). Primary antibodies were added to immunoblots (1:1000 in 2.5% milk in PBST) and incubated overnight at 4 °C with rocking. Immunoblots were washed four times, 15 min each in PBST; horseradish peroxidase–conjugated secondary antibodies were added at a 1:5000 dilution in 2.5% milk in PBST; after a final wash (4 times, 15 min each) in PBST, blots were exposed with SuperSignal Chemiluminescent substrate (Thermo Scientific). The primary antibodies used in this study were as follows: anti-HSF1 (Bethyl) (5, 33) and (10H8, Enzo), anti-CTCF (188408 and 37477, Abcam), anti-HA (Y-11, Santa Cruz), anti-FLAG (M2, Sigma), and anti-GAPDH (6C5, Santa Cruz).

### Statistical analysis

*p*-values were obtained using a Student's *t* test comparing means, using two-tailed, unpaired *t* test for samples with heteroscedastic variance. Error bars shown represent mean ± SEM (standard error of the mean). Reported *p*-values correspond to the following: ∗*p* < 0.05, ∗∗*p* < 0.01, ∗∗∗*p* < 0.001; ns, not significant. Pearson correlation and corresponding *p*-value for the correlation was calculated in MATLAB and assessed with Rosner's or Grubb's tests.

## Data availability

All data supporting the findings of this publication can be found within the supporting information and the primary publication except those noted here. Fingerprinted proteomics data can be found at MassIVE under the dataset MSV000086045. The raw FASTQ files and density normalized BigWig files can be accessed via Gene Expression Omnibus (GEO; https://www.ncbi.nlm.nih.gov/geo/) using the access code GSE155541.

## Conflict of interest

The authors declare that they have no conflicts of interest with the contents of this article.
