# Supplementary material for: Comparative interactomes of HSF1 in stress and disease reveal a role for CTCF in HSF1-mediated gene regulation
Source: J Biol Chem. 2020 Nov 24;296:100097. doi: 10.1074/jbc.RA120.015452 (PMC7948500; doi:10.1074/jbc.RA120.015452)
Supplement: Supplementary file 1 — Figures S1 to S4 [file mmc1.pdf]

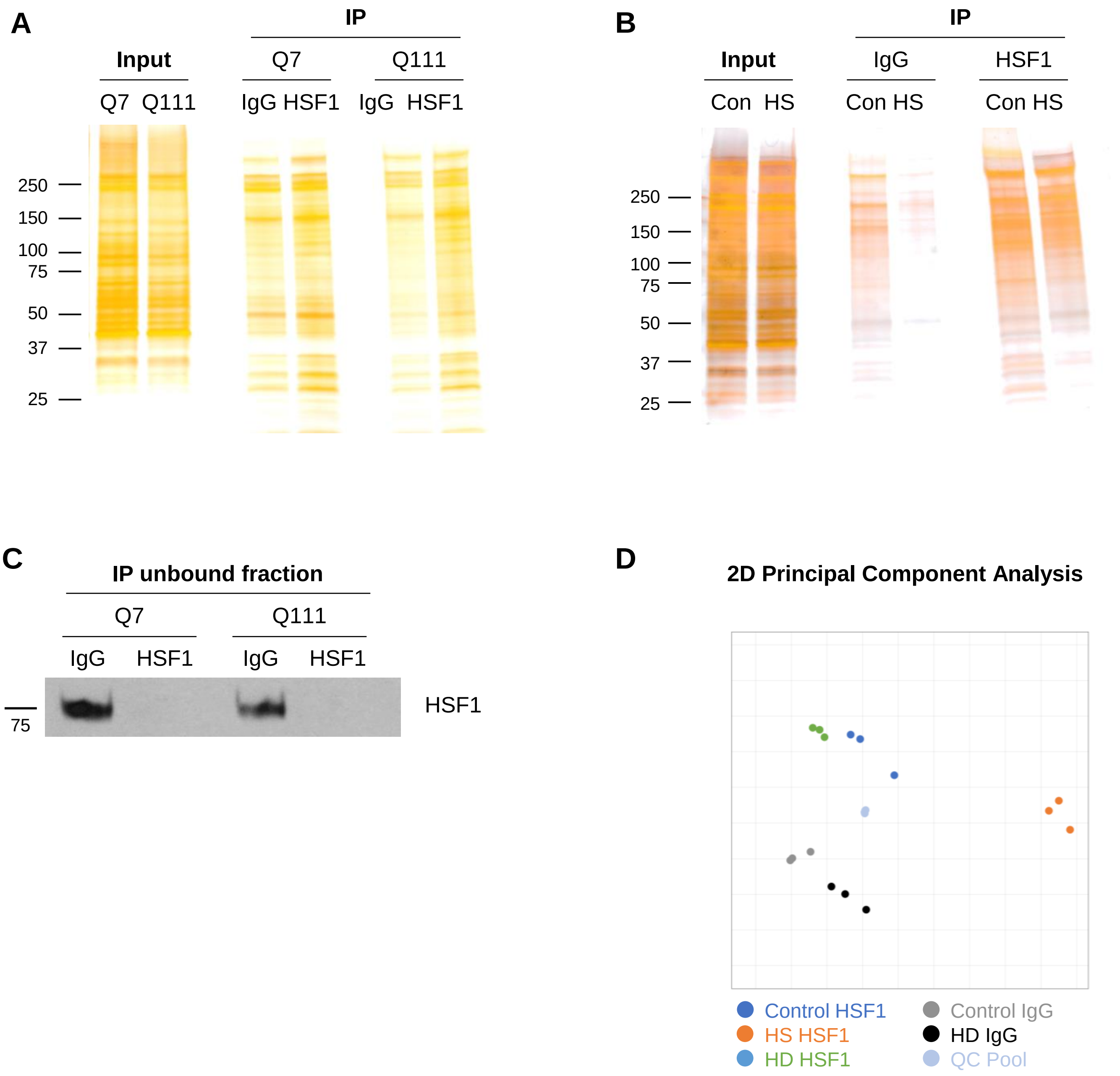

Supporting Information Figure 1

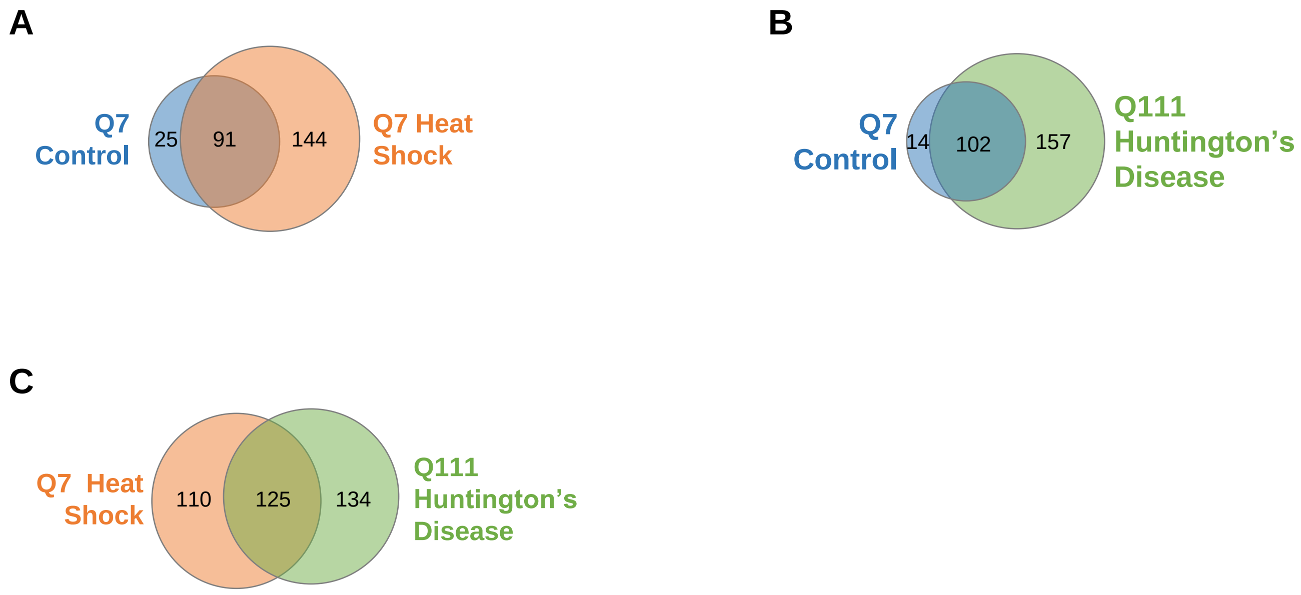

Supporting Information Figure 2

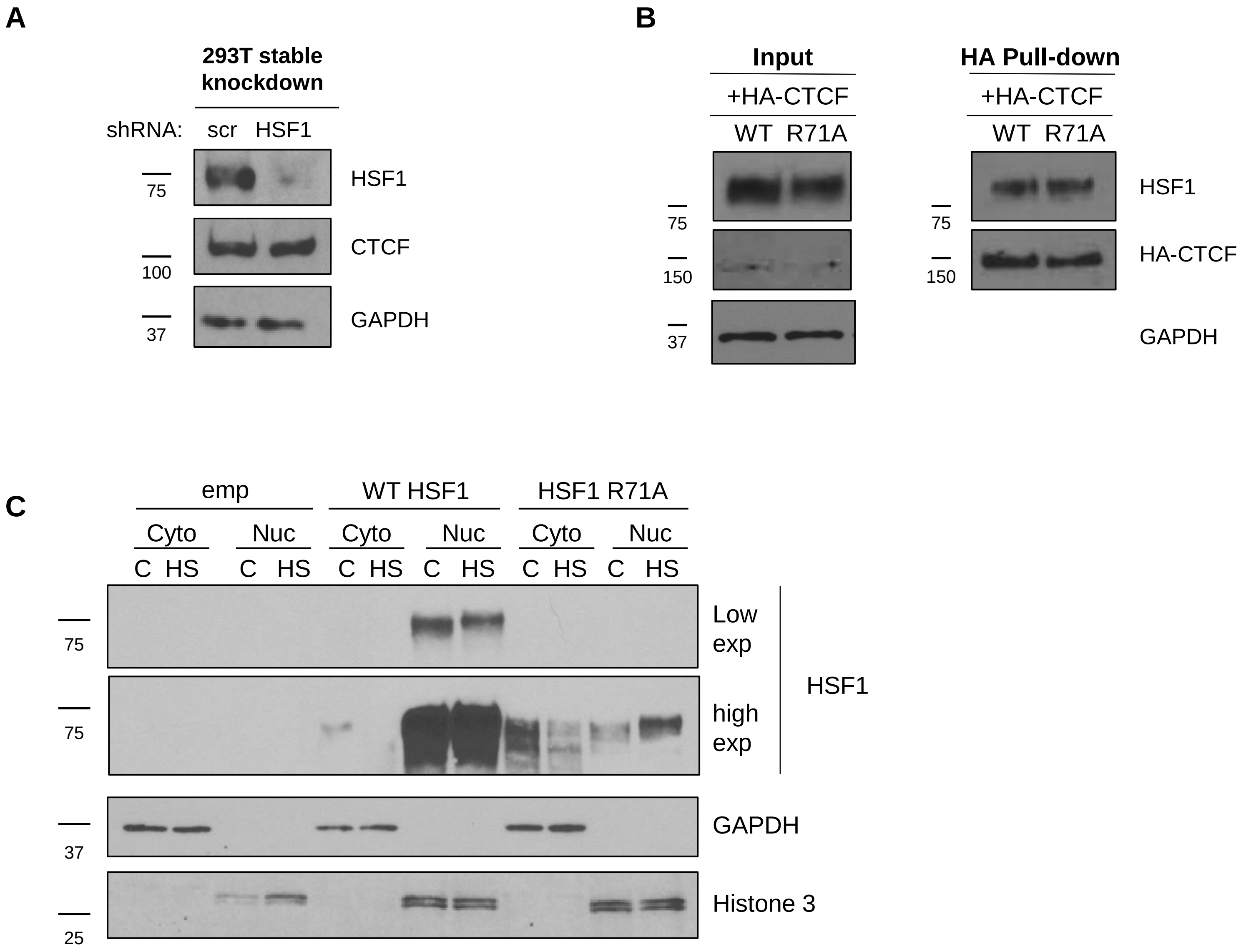

Supporting Information Figure 3

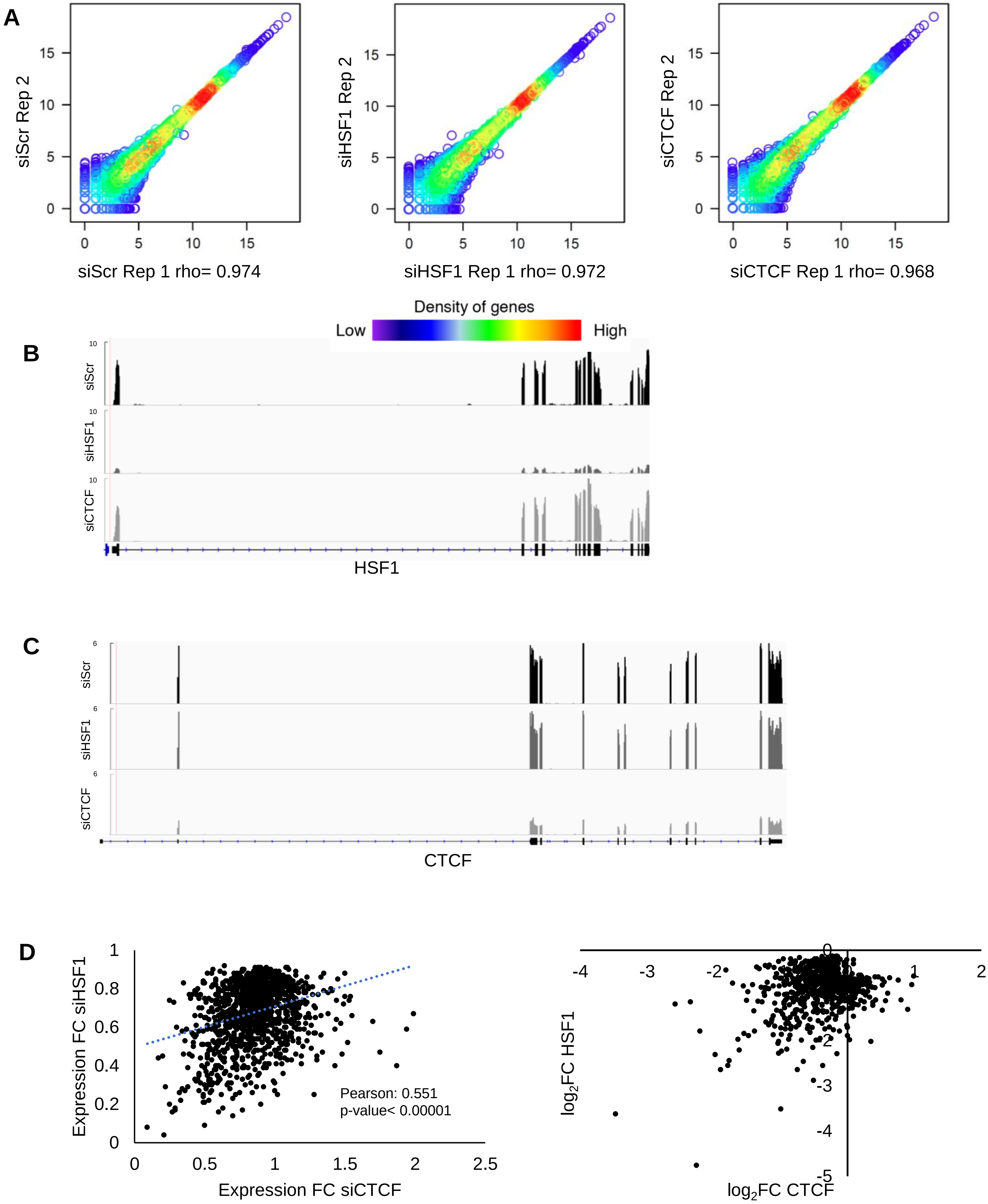

Supporting Information Figure 4

SUPPORTING INFORMATION FIGURE LEGENDS:

Supporting Information Figure 1: HSF1 immunoprecipitation mass spectrometry (A-B) Silver stained SDS-PAGE gels of Control and HD (A) and Con and HS samples (B) after HSF1 or IgG immunoprecipitation from Q7 control, Q7 heat shock, or Q111 HD cells show an enrichment in potential HSF1-interating proteins. (C) Unbound fraction of the IgG or HSF1 IP shows nearly complete HSF1 immunoprecipitation in Control (Q7) and Huntington's Disease (Q111) cells (D) 2D PCA show clustering of biological replicates and separation of samples identified in the different conditions evaluated.

Supporting Information Figure 2: HSF1 unique and shared protein interactors identified in control, heat shock, and Huntington's Disease (A-C) Venn diagram overlap of unique and shared interactions between control and HS (A), control and HD (B), and HS and HD (C); Control conditions are shown in blue, HS in orange, and HD in green.

Supporting Information Figure 3: **Stable knockdown of HSF1, HSF1 DNA-binding deficient mutant interaction with HA-CTCT, and nuclear-cytoplasmic fractionation of HSF1 variants** (A) Stable HSF1 knockdown cells in HEK 293T background compared to shScr stable cell lines show diminished HSF1 protein levels, but no changes in CTCF abundance. (B) $HSF1^{-/-}$ MEFs were transfected with plasmids expressing HA-CTCF and either WT HSF1 or R71A HSF1 and subjected to HA pull-down and immunoblotting with the indicated antibodies. DNA binding of HSF1 is not required for the interaction with CTCF. (C) Nuclear-cytoplasmic fractionation of stable HSF1-knockdown HEK 293T cells shown in (A) transfected with empty vector or a plasmid expressing WT HSF1 or HSF1 R71A. Immunoblots were probed with antibodies against HSF1, GAPDH (cytosolic marker) and Histone H3 (nuclear marker).

Supporting Information Figure 4: Correlation of RNA-sequencing replicates. CTCF and HSF1 do not regulate each other's transcript abundance or splicing (A) Correlation of RNA-sequencing replicates comparing replicate 1 and replicate 2 of each tested condition. These are representative of the correlations observed in all other comparisons (rho>0.95) (B) Integrative Genomics Viewer of fragment per million (FPM) normalized bedgraph files for siScr, siHSF1, and siCTCF from (Fig. 6). siRNA treatment depletes target transcript (HSF1 or CTCF) while untargeted transcript abundance and splicing remain unchanged. (D) Fold Change or log2Fold Change of all detected transcripts in siCTCF or siHSF1-treated cells.

Supporting Information Table 1: Proteins detected in immunoprecipitation mass spectrometry. "Sheet 1: Raw Peptides" shows raw peptide information for each of the 12,497 peptides detected in this study. "Sheet 2: Q7 HSF1 vs Q7 IgG", "Sheet 3: Q7 HS HSF1 vs Q7 IgG", and "Sheet 4: Q111 HSF1 vs Q111 IgG" show raw expression for hit proteins detected in HSF1 IP vs negative control IgG IP for control, heat shock, or Huntington's Disease model cells, respectively. "Sheet 5: Normalized_Hits" shows the normalized counts for the 579 HSF1-interacting proteins that passed stringent criteria outlined in this work (Fold change > 2 for HSF1/IgG IP, $p<0.05$, protein teller probability > 0.8). Proteins from "Normalized_Hits" sheet can only be compared for conditions in which they were statistically enriched over IgG (see Sheets 2-4, Fig. 3, and Supporting Information Table 2 for these protein categories).

Supporting Information Table 2: Unique HSF1 interacting proteins identified in Control, Heat Shock, and Huntington's Disease. Underlined proteins have previously been reported to interact with HSF1 (See Fig. 2e for references).

Supporting Information Table 3: RNA sequencing data. Transcript abundance as measured by RNA sequencing for cells treated with CTCF and HSF1 siRNA.
