# Supplementary material for: Comparative interactomes of HSF1 in stress and disease reveal a role for CTCF in HSF1-mediated gene regulation
Source: J Biol Chem. 2020 Nov 24;296:100097. doi: 10.1074/jbc.RA120.015452 (PMC7948500; doi:10.1074/jbc.RA120.015452)
Supplement: Supplementary file 3 — Table S2 [file mmc3.pdf]

Control	Heat Shock				Huntington's Disease			
ARGI1	AACS	HEAT3	PRS6B	RS27A	ABCE1	EMAL4	MAP4	RM47
ASAP2	ABCF3	IF4A1	PRS7	SDCB1	ABCF1	ERF1	MARK2	RSBN1
DESP	ACADS	IF4G2	PRS8	SMTN	AKAP1	EXOS4	MARK3	RT35
LEG3	ACADV	ILK	PSA1	SNX9	ANM5	EXOS5	MATR3	SAP18
LRCH3	ACTZ	IMA1	PSA2	SYTC	ASCC1	EXOS6	MET13	SDPR
NEST	ANXA4	IMB1	PSA3	TBA1A	ASCC2	EXOS7	MGN	SK2L2
PKP1	ANXA5	IMPCT	PSA4	TBB6	ASCC3	EXOS9	MK67I	SKP1
PLAK	ANXA6	IPO4	PSA5	TCTP	ATX2	EXOSX	MLTK	SMAG1
SKT	ANXA7	IPO5	PSA6	TERA	CC124	FA83D	MRCKB	SMC5
SMG1	AP1B1	IPO7	PSA7	TNPO1	CE170	FXR1	MRT4	SNUT1
	AP3B1	IPO9	PSB1	TRAP1	CH033	GNL3L	NCBP1	SPAS2
	ATPB	IQGA1	PSB2	TRI56	CKAP2	GTF2I	NCBP2	SPB1
	BZW1	LPPRC	PSB4	UBA1	DCA13	GTPB1	NEMF	SRPK1
	C1TC	MMS19	PSB5	XPO5	DDX10	HERC6	NFX1	SRPK2
	CLIC1	MTAP2	PSB6	XPOT	DDX27	HNRL2	NOG1	SRRT
	COPB	MYO1C	PSD11	XPP1	DDX31	HNRPC	NOP58	SYGP1
	COPB2	NAT10	PSD12		DDX3X	HNRPU	PABP1	TBL3
	COPG1	NEDD4	PSD13		DDX54	IF2A	PAR12	TRIP4
	DDX47	NMNA1	PSDE		DHX29	IF2B	PESC	TRM6
	DIC	NOL6	PSMD1		EBP2	IF2G	PRP17	TSR1
	E41L2	NUP93	PSMD2		EI2BD	IF2P	PRP8	TUT7
	ECHA	ODO1	PSMD3		EI2BE	IF4A3	PWP2	U520
	EF1G	P5CS	PSMD4		EIF2D	IF4B	RALY	U5S1
	EF2	PALLD	PSMD6		EIF3A	KANK2	RASL2	UTP18
	EM55	PDC6I	PSMD7		EIF3B	KIF1C	RBM28	UTP4
	FACR1	PEX14	PSMD8		EIF3C	KIF2A	RBM8A	WDR3
	GCN1	PPCE	RABP1		EIF3D	LAR4B	RFC1	WDR46
	GSTO1	PRS10	RINI		EIF3F	LARP4	RFC5	WDR61
	HABP4	PRS4	RPB1		EIF3L	LARP7	RIOK1	XRCC5
	HCD2	PRS6A	RPB3		EIF3M	MAP2	RL7L	

Supporting Information Table 2
